# Axin2-expressing cells differentiate into reparative odontoblasts via autocrine Wnt/β-catenin signaling in response to tooth damage

**DOI:** 10.1038/s41598-017-03145-6

**Published:** 2017-06-08

**Authors:** Rebecca Babb, Dhivya Chandrasekaran, Vitor Carvalho Moreno Neves, Paul T. Sharpe

**Affiliations:** Centre for Craniofacial and Regenerative Biology, Dental Institute, Kings College London, London, UK

## Abstract

In non-growing teeth, such as mouse and human molars, primary odontoblasts are long-lived post-mitotic cells that secrete dentine throughout the life of the tooth. New odontoblast-like cells are only produced in response to a damage or trauma. Little is known about the molecular events that initiate mesenchymal stem cells to proliferate and differentiate into odontoblast-like cells in response to dentine damage. The reparative and regenerative capacity of multiple mammalian tissues depends on the activation of Wnt/β-catenin signaling pathway. In this study, we investigated the molecular role of Wnt/β-catenin signaling pathway in reparative dentinogenesis using an *in vivo* mouse tooth damage model. We found that Axin2 is rapidly upregulated in response to tooth damage and that these Axin2-expressing cells differentiate into new odontoblast-like cells that secrete reparative dentine. In addition, the Axin2-expressing cells produce a source of Wnt that acts in an autocrine manner to modulate reparative dentinogenesis.

## Introduction

The complex structural composition of adult mammalian teeth provides a hard-outer barrier that protects the inner dental pulp from being exposed to the external environment. This barrier consists of two mineralised tissues, an outer layer of enamel and an inner thicker layer of dentine. If this mineralised barrier is damaged due to a trauma or dental caries, the dental pulp has the ability to produce a form of tertiary dentine called reactionary dentine by stimulation of specialised cells known as primary odontoblasts located at the periphery of the pulp chamber that are responsible for dentine secretion^1–3^. In non-growing teeth, such as molars, primary odontoblasts can be lost if the dentine is breeched and the pulp is exposed. A second generation of odontoblast-like cells that originate from mesenchymal stem cells (MSCs) in the pulp can replace lost primary odontoblasts. The newly differentiated odontoblast-like cells secrete a form of tertiary dentine called  reparative dentine, (also referred to as a dentine bridge) to seal the site of exposure and maintain pulp vitality. Continuously growing teeth, such as rodent incisors produce new dentine throughout life as an adaptation to the self-sharpening at their tips^4^. The continuous turnover of odontoblasts and pulp cells in the incisor is supported by mesenchyme stem cells (MSCs) that reside in a neurovascular niche at their proximal ends^5–7^. Unlike incisors, little is known about the molecular events that initiate MSCs to proliferate and differentiate into odontoblast-like cells in response to dentine damage in molars. The canonical Wnt/β-catenin signaling pathway is important for stem cell renewal, proliferation and differentiation^8, 9^. Activation of Wnt/β-catenin signaling causes β-catenin to accumulate in the cytoplasm and translocate to the nucleus where it activates downstream target genes. During tooth development, Wnt/β-catenin signaling is required at various stages of tooth morphogenesis^10^. Wnt responsive genes, such as Axin2 are expressed in differentiating odontoblasts, implicating Wnt/β-catenin signaling in odontoblast development and maturation^11–13^. Inactivation of β-catenin expression during tooth development leads to the disruption of odontoblast differentiation and root formation whereas, the overexpression of β-catenin results in excessive dentine production from mature odontoblasts^14, 15^. Limited information is available concerning the importance of Wnt/β-catenin signaling in the generation of new odontoblast-like cells in postnatal teeth. The reparative and regenerative capacity of multiple mammalian tissues depends on the activation of Wnt/β-catenin signaling pathway and both epithelial and mesenchymal stem cells depend on this pathway being active to drive tissue renewal and repair^16^. Several studies have shown that tissue specific stem cells involved in tissue regeneration and repair can be identified by genetically-labelled Wnt responsive genes^17–21^. It has previously been shown that elevated Wnt signaling  enhances reparative dentinogenesis in Axin2^LacZ/LacZ^ mice^22^. We have shown that delivery of small molecule inhibitors of GSK3 activity (Wnt/β-catenin signaling antagonists) directly to exposed pulps promotes the production of reparative dentine *in vivo* ^23^. In this study, we take advantage of genetically-modified mice to investigate the molecular role of Wnt/β-catenin signaling in the reparative dentinogenesis process. Our study has revealed that Axin2-expressing cells differentiate into new odontoblast-like cells that secrete reparative dentine. In addition, Axin2-expressing cells produce a source of Wnt ligands that acts in an autocrine manner to modulate reparative dentinogenesis.

## Methods

### Mouse and animal information

All animals used in this study were handled in accordance with UK Home Office Regulations project license 70/7866 and personal license I6517C8EF. Experimental procedures were approved by the King’s College Ethical Review Process. Axin2^LacZ/LacZ^, Axin2^CreERT2, Rosa26-mTmG flox/+ (fl/+)^ and pCagCreERT2 Wntless(Wls)^flox-flox (fl/fl)^ were obtained from the Jackson Laboratory. TCF/Lef:H2B-GFP reporter mice were a kind gift from Anna-Katerina Hadjantonakis^24^. To induce genetic recombination, adult mice (6 weeks) were injected intraperitoneally with tamoxifen dissolved in corn oil/10% (vol/vol) ethanol, corresponding to specific doses per gram body weight (0.1 mg of tamoxifen per gram of body weight). Axin2^CreERT2^ mice received one dose of tamoxifen or corn oil (WT) on the day of tooth damage and another two doses over the next two consecutive days. Wls^fl/fl^ and Axin2^CreERT2, Rosa26-mTmG fl/+^; Wls^fl/fl^ mice received one dose of tamoxifen or corn oil (WT) over three consecutive days and tooth damage was performed 5 days post last tamoxifen or corn oil injection.

### Tooth damage protocol

Mice were anaesthetised with a solution made with Hypnorm (Fentanyl/fluanisone - VetaPharma Ltd.), sterile water and Hypnovel (Midazolam - Roche) in the ratio 1:2:1 at the rate of 10 ml/kg intraperitonially. The oral cavity was opened with a mouth retractor to expose the molars. The superior first molars were cleaned using a sterile cotton plug soaked in phosphate buffered saline (PBS). A cavity was drilled in the centre of the superior first molar using a carbide bur (FG^1/4^) coupled to a high speed hand piece (Kavo Super Torque LUX 2 640B). Drilling was stopped when the pulp was visible under the dentine roof and a 27 G^3/4^ needle was used to expose the pulp chamber. Mineral Trioxide Aggregate (MTA; Maillefer, Dentsply) was applied to the exposed pulp and the cavity was sealed with glass ionomer Ketac™ Cem radiopaque (3 M ESPE). Post-op, the mice were given Vetergesic (Buprenorphine – Ceva) at the rate of 0.3 mg/kg intraperitonially as analgesic. The animals were sacrificed after at various time points post-damage.

### Dental pulp extraction

Dental pulp tissue was extracted from superior first molars collected from CD-1 P21 mice according to the experiment time course post-damage. A 21G needle was used as an elevator to extract teeth from the alveolar bone. The extracted teeth were placed in ice cold PBS and a 23 scalpel blade was used to separate the tooth at the crown-root junction to expose the pulp chamber. The pulp was gently removed from the pulp chamber and the root canal using a 0.6 mm straight tip tweezer. The pulp was stored in RNAlater (Sigma) at −80 °C. For each condition, dental pulp tissue was pooled from at least 10 teeth and repeated in triplicate.

### Real-time qPCR analysis

Total RNA was extracted from the dental pulp using TRIzol (Invitrogen) as recommended by the manufacturer’s instructions. The RNA was reverse transcribed using random primers (M-MLV Reverse Transcriptase kit, Promega) according to the manufacturer’s instructions. Gene expression was then assayed by real-time qPCR using Kappa Syber Fast (Kappa Biosystems) on a Rotor-Gene Q cycler (Qiagen) system. Beta-actin primers (Forward - GGCTGTATTCCCCTCCATCG, Reverse - CCAGTTGGTAACAATGCCTGT) were used for the housekeeping gene, Axin2 primers (Forward -TGACTCTCCTTCCAGATCCCA, Reverse -TGCCCACACTAGGCTGACA) were used as the read-out of Wnt activity and for Gpr177 (Wls) expression, Forward – TCTAATGGTGACCTGGGTGTC and Reverse – TTCCAGCTCAGTGCCATACC primers were used. Reactions were performed in triplicate and relative changes to housekeeping gene were calculated by the 2^−^^ΔΔC^_T_ method.

### Tissue processing and histological staining

Teeth were fixed in 4% paraformaldehyde (PFA) for 24-hours at 4 °C, washed with PBS and decalcified in 19% EDTA, pH 8 for 4 weeks. Decalcified teeth were dehydrated through a graded series of ethanol, cleared in xylene and infused with wax at 60 °C in a Leica ASP300 tissue processor. Samples were embedded in wax and 8 μm sections were cut using a microtome (Leica RM2245, blade angle 5°). Sections were mounted on Trubond^TM^ 380 slides (Electron Microscopy Sciences). For Masson’s Trichrome staining, sections of adult teeth were deparaffinised in Neo-Clear and rehydrated through a series of graded ethanol. Sections were stained with Weigert’s Haematoxylin for 10 minutes, followed by 1% Biebrich-Scarlet-Acid Fuchsin solution for 15 minutes, differentiated in a mix of 5% phosphomolybdic acid and 5% phosphotungstic acid for 15 minutes and stained with 2.5% Aniline Blue solution for 10 minutes. Following staining, sections were differentiated in 1% acetic acid, dehydrated through 90% and 100% ethanol, cleared in Neo-Clear and permanently mounted in Neo-Mount.

### Immunohistochemistry

Sections of adult teeth were deparaffinised in xylene and rehydrated in graded ethanol. To reduce endogenous peroxidase activity, sections were quenched with 3.5% hydrogen peroxide in PBS for 5 minutes and blocked with 10% goat serum in PBS with 0.1% Tween20 (PBST). No antigen retrieval was performed. The Primary antibodies were applied overnight at 4 °C. Proliferating cell nuclear antigen (PCNA, Abcam, ab18197) and green fluorescent protein (GFP, Abcam, ab13970) antibodies were used at 1:200 and 1:500, respectively in PBST containing 5% goat serum. After washing the sections with PBST they were incubated with appropriate biotinylated secondary antibody, then horseradish peroxidase (HRP)-conjugated streptavidin-biotin antibody and washed with PBST. Immunoreactivity was visualised with ImmPACT DAB HRP Substrate (Vector Laboratories) or MenaPath green chromogen kit (Bio SB). Sections were counterstained with hematoxylin, dehydrated in graded ethanol, cleared in xylene and mounted in DPX. Sections were viewed in light-field using a Zeiss microscope (Axioskope 2 plus) and captured with an AxioCam HRC (Zeiss) using Axiovision software. For immunofluorescent co-staining, PCNA and GFP antibodies were used at 1:200 and 1:500, respectively in PBST containing 5% goat serum. After washing the sections with PBST they were incubated with Alexa Fluor® 647 to detect GFP and Alexa Fluor® 568 to detect PCNA and counterstained with Hoechst (40 μg/ml) in PBS before mounting with Citifluor. Immunofluorescence was visualised with a Leica TCS SP5 laser confocal microscope.

### *In situ* hybridisation and Immunofluorescence

*In situ* hybridisation for dentine sialo-phosphoprotein (*Dspp)* was performed on paraffin sections following standard procedures under RNase-free conditions^25^. *Dspp* was detected using TSA biotin system (PerkinElmer) in combination with the TSA Plus Cyanine 3.5 detection kit (PerkinElmer). After *in situ* hybridisation, slides were blocked with 10% goat serum in PBST, incubated with anti-GFP antibody (Abcam, ab13970, 1:200) in PBST with 5% goat serum overnight at 4 °C. Sections were washed with PBST, incubated with Alexa Fluor® 647 secondary antibody and counterstained with Hoechst (40 μg/ml) in PBS before mounting with Citifluor. Immunofluorescence was visualised with a Leica TCS SP5 laser confocal microscope.

### Statistical Analysis

A two tailed unpaired Student’s *t*-test using GraphPad prism software was used to determine significance, a *P*-value < 0.05 was considered statistically significant. At least four independent experiments were performed for statistical analysis of dental pulp tissues described in the figure legends. Statistical data was presented as mean ± s.e.m.

### Data Availability

The datasets generated during and/or analysed during the current study are available from the corresponding author on reasonable request.

## Results

### Time course of reparative dentinogenesis in tooth damage model

To determine the timescale of reparative dentinogenesis in our model, damaged teeth were stained with Masson’s Trichrome to visualise dentine production, *in situ* hybridisation detection of *Dspp* gene was performed to identify odontoblasts^26, 27^ and immunohistochemical staining PCNA was preformed to detect proliferating cells.

**Figure 1:**
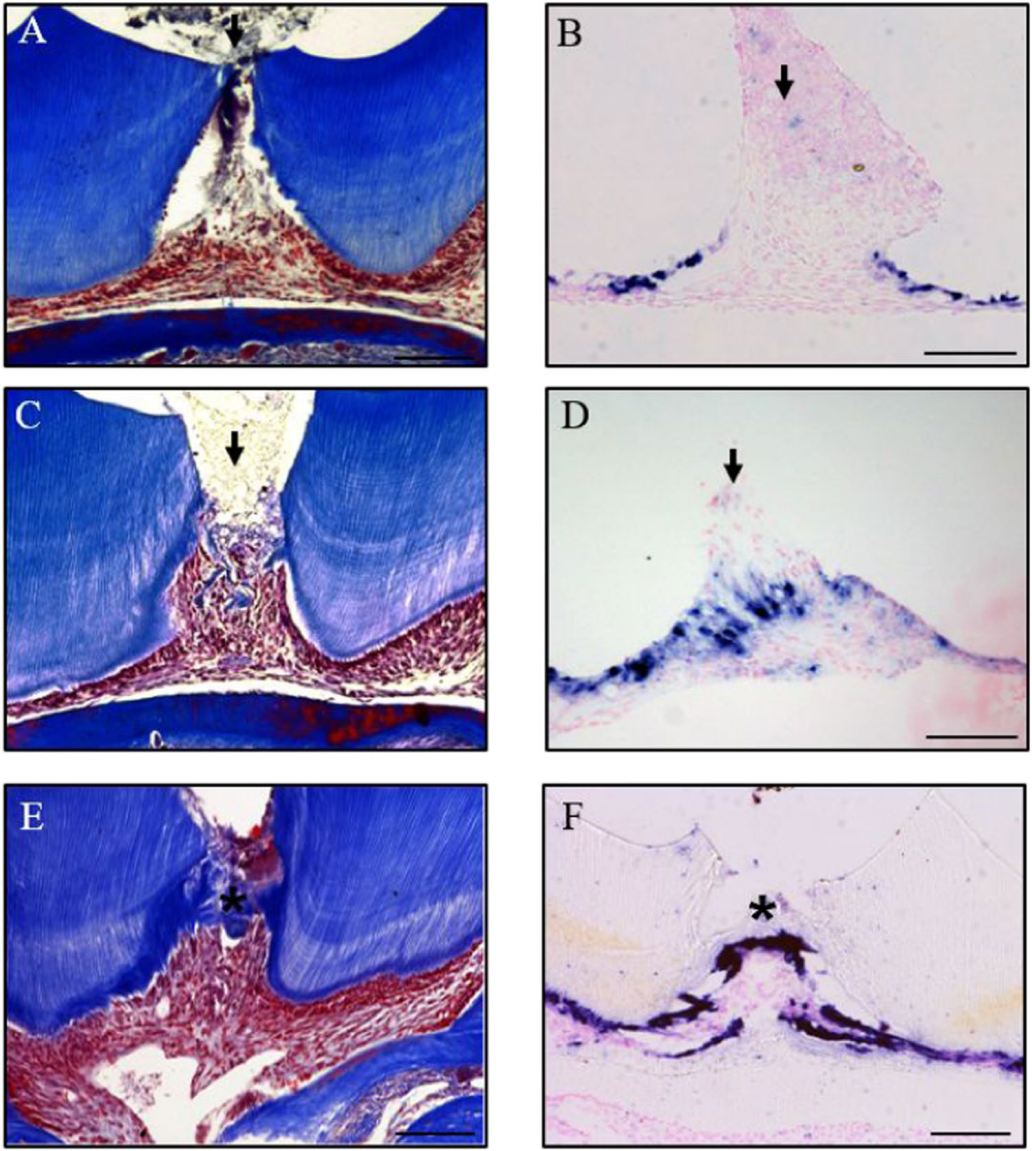
Time course of reparative dentinogenesis in molar tooth damage model. Masson’s Trichrome staining of a superior first molar 1 day post-damage (**A**) and *in situ* hybridisation analysis of *Dspp* expression in a superior first molar 1 day post-damage (**B**). Masson’s Trichrome staining of a superior first molar 5 days post-damage (**C**) and *in situ* hybridisation analysis of *Dspp* expression in a superior first molar 5 days post-damage (**D**). Masson’s Trichrome staining of a superior first molar 14 days post-damage (**E**) and *in situ* hybridisation analysis of *Dspp* expression in a superior first molar 14 days post-damage (**F**). All damage was performed in CD-1 mice and representative sagittal sections are shown from four independent experiments. Scale bars are equivalent to 100 μm and an arrow indicates the site of damage.

**Figure 2:**
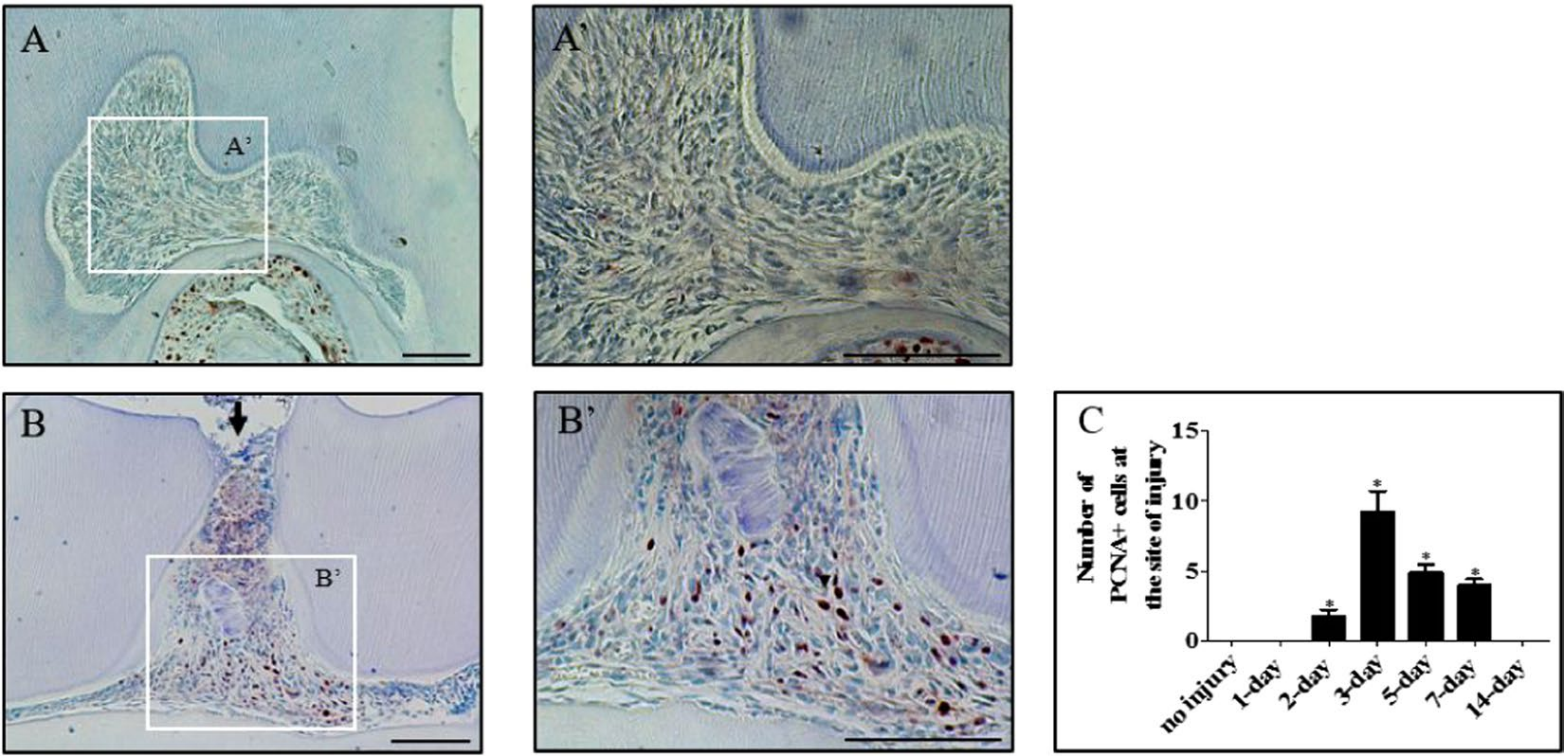
Pulp cells proliferate in response to damage. Immunohistochemical staining of proliferating cells with a PCNA antibody in an undamaged molar at low (**A**) and high magnification (A’). Immunohistochemical staining of proliferating cells with a PCNA antibody in a damaged superior first molar 3 days post-damage at low (**B**) and high magnification (B’). All damages were performed in CD-1 mice and representative sagittal sections are from four independent experiments. Scale bars are equivalent to 100 μm, an arrow indicates a pulp exposure. Quantification of the PCNA positive cells (**C**) was performed on three high-powered fields on at least four specimens per group, *p = <0.05.

*In situ* hybridisation detection of *Dspp* showed primary odontoblasts located at the periphery of the dental pulp expressed *Dspp*, however no *Dspp* expression was detected at the site of exposure at 1 day post-damage, due to loss of local primary odontoblasts (Fig. 1A,B). *Dspp* expression was observed at the site of exposure 5 days post-damage and some reparative dentine was visible (Fig. 1C,D). By 14 days post-damage, a dentine bridge was formed and *Dspp* expression is localised underneath the dentine bridge (Fig. 1E,F). Immunohistochemical detection of PCNA in undamaged molars showed that there is little if any proliferation in the pulp chamber (Fig. 2A). When molars were damaged, dental pulp cells began to proliferate underneath the site of damage by 2 days post-damage, with proliferation peaking at 3 days and returning to resting levels by 14 days post-damage (Fig. 2B,C).

Collectively, these data demonstrate that cells of the dental pulp proliferate then differentiate into new odontoblast-like cells that secrete reparative dentine in response to damage.

### Wnt/β-catenin signaling is activated in proliferating cells in response to tooth damage

We next wanted to investigate the role of Wnt/β-catenin signaling in reparative dentinogenesis process. Axin2^LacZ^ reporter mice have been widely used to visualise Wnt active cells *in vivo* ^22^. We observed diffusely stained Axin2 positive cells scattered under the site of exposure in damaged teeth from Axin2^LacZ^ reporter mice (Supplementary Figure 1). It was not possible to assess if Axin2 cells were proliferating in damaged teeth from Axin2^LacZ^ reporter mice as the LacZ staining was not compatible with immunohistochemistry. We thus used TCF/Lef:H2B-GFP reporter mice that allow the visualisation of Wnt active cells since β-catenin is a transcriptional cofactor for TCF/Lef that is upstream regulator of Axin2.

**Figure 3:**
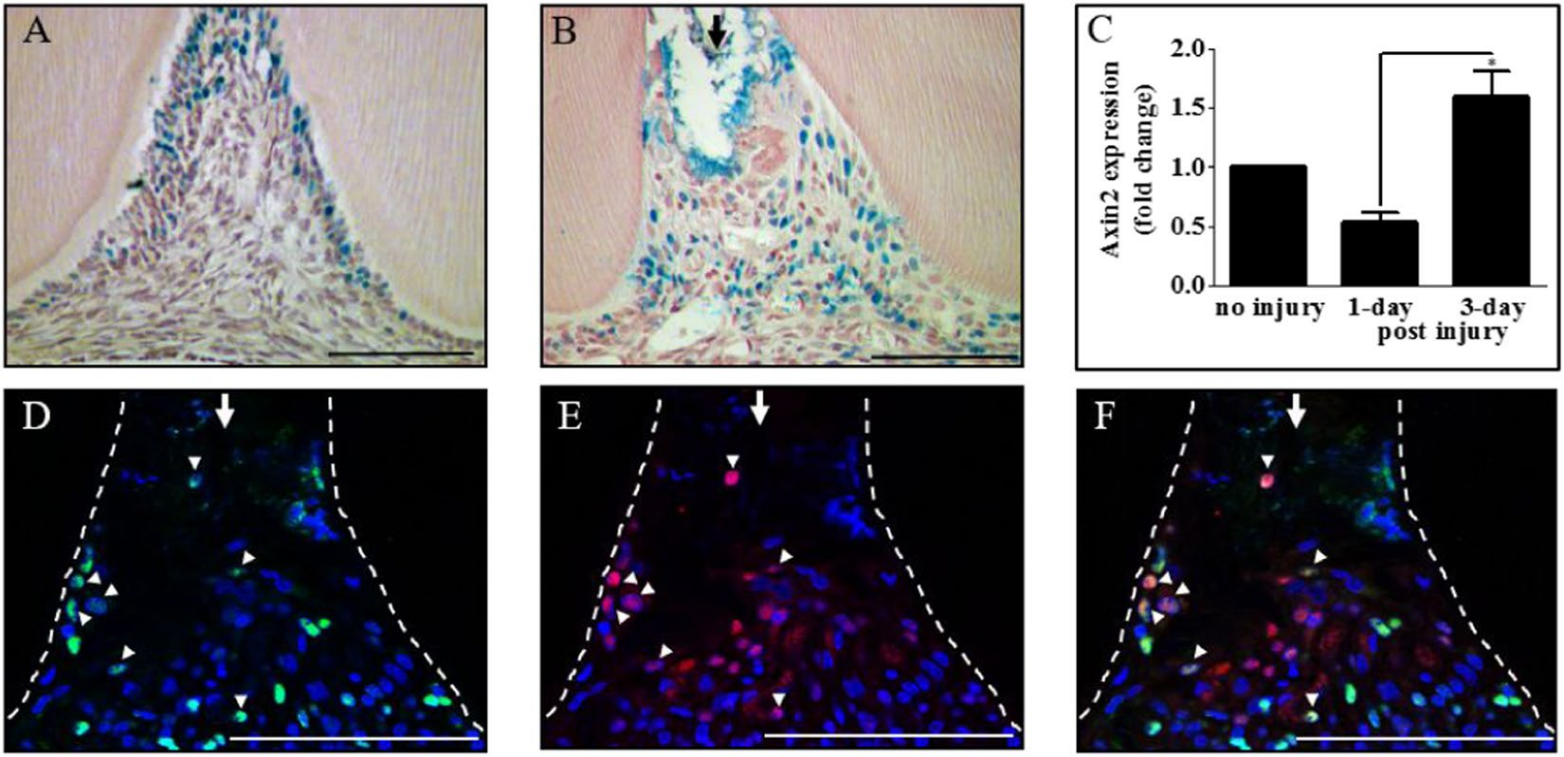
Wnt/β-catenin signaling is activated in proliferating cells in response to tooth damage. Immunohistochemical staining of TCF/Lef (Wnt reporter) cells with a GFP antibody in an undamaged (**A**) and damaged superior first molar 3 days post-damage (**B**) from TCF/Lef:H2B-GFP reporter mice. Real-time qPCR analysis of Axin2 gene expression in dental pulp tissue extracted from undamaged and damaged maxillary first molars (**C**), n = 4, *p = <0.05. Immunohistochemical staining of TCF/Lef:H2B-GFP cells with a GFP antibody (**D**), proliferating cells with a PCNA antibody (**E**) and merged image (**F**) in a damaged superior first molar 3 days post-damage from TCF/Lef:H2B-GFP reporter mice. Representative sagittal sections are shown from four independent experiment’s. Scale bars are equivalent to 100 μm. The dentine-pulp interface is outlined by a white dashed line drawn from the light field image. An arrow indicates pulp exposure and arrow heads indicate examples of double stained cells.

Immunohistological staining of undamaged molars from TCF/Lef:H2B-GFP mice with a GFP antibody showed that some primary odontoblasts had strong staining at the periphery of the pulp cusp (Fig. 3A). Following damage, Wnt active cells at the periphery of the top of the cusp were lost due to pulp exposure and Wnt active were now detected throughout the pulp tissue under the site of exposure 3 days post-damage (Fig. 3B). Furthermore, real-time qPCR analysis of Axin2 expression from extracted dental pulps showed that Axin2 expression was initially reduced, presumably as a result of the loss of Wnt active primary odontoblasts 1 day post-damage, then significantly increased by 3 days post-damage (Fig. 3C). Immunofluorescent co-staining of damaged molars from TCF/Lef:H2B-GFP mice with GFP and PCNA antibodies, showed that some Wnt positive cells were proliferating 3 days post-damage (Fig. 3D–F).

### Odontoblast-like cells are descendants of Wnt active cells

To investigate the fate of Wnt active cells we used Axin2^CreERT2^^;^^Rosa26 mT-mG^ ^flox/+^ mice to lineage trace the progeny of Axin2-expressing cells. In these mice, cells that express Axin2 are permanently labelled with GFP during the tamoxifen administration period, allowing fate mapping of labelled cells and their descendants. We administered tamoxifen immediately after tooth damage and for the next two days meaning only cells that express Axin2 in this period will be permanently labelled.

**Figure 4:**
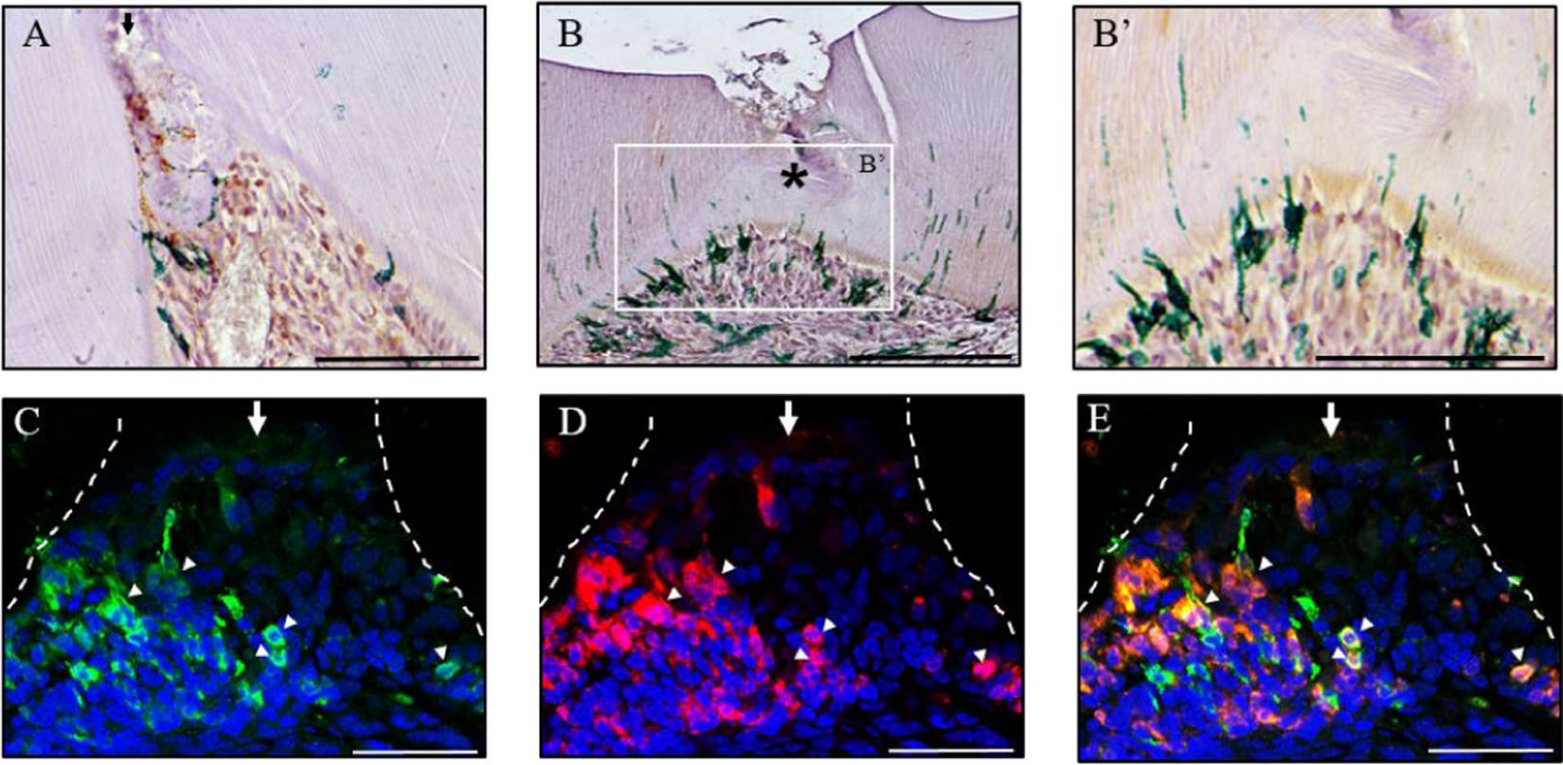
Odontoblast-like cells are descendants of Wnt active cells. Immunohistochemical staining of Axin2-expressing cells with GFP in a damaged superior first molar 3 days post-damage (**A**) and 14 days post-damage at low (**B**) and high magnification (B’). Immunofluorescent staining of Axin2-expressing cells with GFP (**C**), *in situ* hybridisation analysis of *dspp* expression (**D**) and merged image (**E**) of damaged superior first molar from Axin2^CreERT2; Rosa26-mT-mG flox/+^ mice 5 days post-damage. Representative sagittal sections are shown from four independent experiment’s. Scale bars are equivalent to 100 μm. The dentine-pulp interface is outlined by a white dashed line drawn from the light field image. An arrow indicates pulp exposure; arrow heads indicate examples of double stained cells and asterisk indicates the formation of a dentine bridge.

Immunohistochemical detection of GFP showed a few Axin2-labelled cells at the site of damage 3 days post-damage (Fig. 4A) and an increase in the number of Axin2-labelled cells were detected 14 days post-damage beneath the dentine bridge (Fig. 4B,B’). This data suggests that a population of pulp cells are expressing Axin2 in response to damage and these labelled cells are undergoing a proliferative expansion. Furthermore, some of the Axin2-expressing cells were in close association with the newly formed dentine bridge and had characteristic morphology of odontoblasts. These data demonstrate that Axin2-expressing cells can differentiate into reparative odontoblast-like cells. Moreover, immunofluorescent staining of Axin2-labeled cells with GFP and co-detection of *Dspp* by *in situ* hybridisation, revealed that some Axin2-labeled cells co-expressed *Dspp* 5 days post-damage underneath the site of exposure (Fig. 4C–E). This data suggests that reparative odontoblasts are descendants of Axin2-expressing cells.

### Inhibition of Wnt signaling impairs reparative dentinogenesis

**Figure 5:**
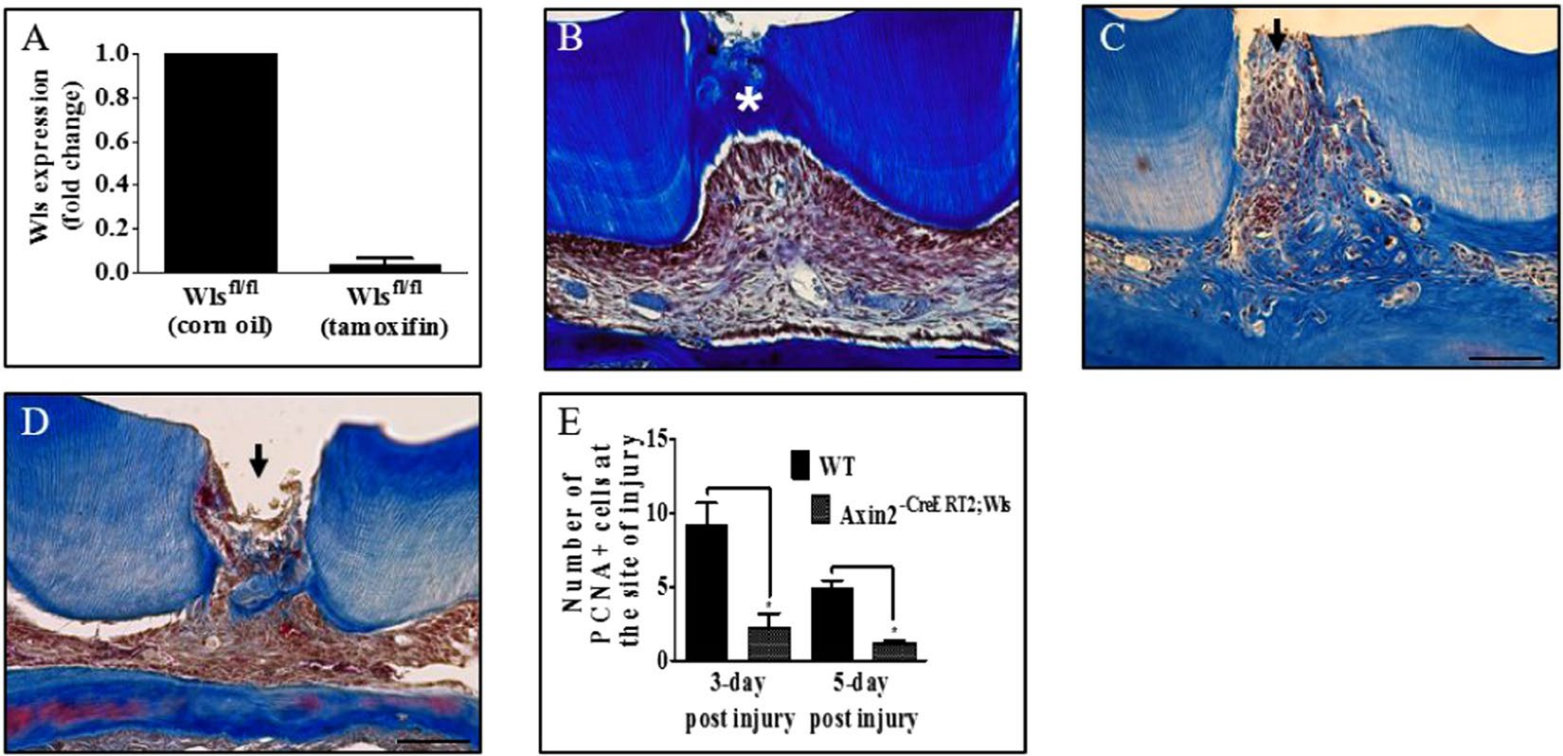
Inhibition of Wnt signaling in Axin2-expressing cells impairs reparative dentinogenesis. Real-time qPCR analysis of Wls gene expression in dental pulp tissue extracted from WT and Wls^fl/fl^ mice teeth 5 days post-damage (**A**) Masson’s Trichrome staining of a damaged superior first molar from WT mice (**B**), Wls^fl/fl^ mice (**C**) and Axin2^CreERT2^;Wls^fl/fl^ mice 14 days post-damage (**D**). Representative sagittal sections are shown, scale bars are equivalent to 100 μm, an arrow indicates the site of damage and an asterisk indicates a dentine bridge. Quantification of PCNA positive cells in damaged superior first molar from WT and Axin2^CreERT2^; Wls^fl/fl^ mice post-damage (**E**), n = 4, *p = <0.05.

It has previously been shown that elevating Wnt signaling enhances reparative dentinogenesis *in vivo* ^22^. This was shown using Axin2^LacZ/LacZ^ mice that are a model for elevated Wnt/β-catenin signaling since these mice lack functional copies of both Axin2 alleles, a negative regulator of Wnt signaling. We also confirmed these findings using our molar damage model and additionally compared reparative dentine formation by elevated Wnt/β-catenin activity with that from a commonly used clinical capping agent, MTA (mineral trioxide aggregate ) (Supplementary Figure 2). In addition, we investigated whether impairing Wnt/β-catenin signaling affected reparative dentinogenesis. Gpr177 (Wls)^flox/flox (fl/fl)^ mice do not express the Wls gene that encodes a sorting receptor required for Wnt secretion, thus cells cannot release Wnts to activate Wnt/β-catenin signaling. Real-time qPCR confirmed that the expression of the Wls gene was drastically reduced in the dental pulp tissue of Wls^fl/fl^ mice 5 days post-tamoxifen compared to WT mice (Fig. 5A). Masson’s Trichrome staining of damaged molars from Wls^fl/fl^ showed that reparative dentinogenesis does not occur in the absence of Wnt signaling since no dentine bridge was formed in response to damage by 14 days post-damage compared to WT (Fig. 5B,C).

However, we did observe resorption pits in the pulp chamber and the presence of TRAP positive cells in damaged molars, suggesting the presence of osteoclasts (Supplementary Figure 3). We also observed resorption pits on the roots of undamaged molars (Supplementary Figure 3). To overcome this pathology, we crossed Wls^fl/fl^ mice with Axin2^CreERT2^ mice to specifically delete Wls in Axin2-expressing cells, thus only preventing these cells from secreting Wnts. Masson’s Trichrome staining of damaged molars from Axin2^CreERT2^; Wls^fl/fl^ mice showed that reparative dentinogenesis was severely impaired 14 days post-damage (Fig. 5D). No resorption pits were observed in undamaged or damaged teeth of Axin2^CreERT2^; Wls^fl/fl^ mice. Additionally, cell proliferation in response to pulp exposure was significantly reduced in damaged molars of Axin2^CreERT2^; Wls^fl/fl^ mice compared to WT 14 days post damage (Fig. 5E).

## Discussion

To study the molecular mechanisms of reparative dentinogenesis, we established a controlled molar-damage model *in vivo*. The damage (100 μm in diameter) was created occlusally in the centre of the superior first molar crown to expose the dental pulp tissue with subsequent capping with mineral trioxide aggregate (MTA) and glass ionomer restoration. We used MTA as it is a biocompatible material that releases calcium ions that are thought to stimulate reparative dentine formation^28–30^. In endodontic procedures MTA is commonly used in vital pulp therapy to treat exposed dental pulp^31^. In our model, we observed robust proliferation and differentiation of a population of cells into new odontoblast-like cells that secrete reparative dentine at the site of damage.

To investigate the role of Wnt/β-catenin signaling in reparative dentinogenesis we took advantage of genetic mice models. Axin2^LacZ^ and TCF/Lef:H2B-GFP reporter mice and real-time qPCR analysis of Axin2 expression, revealed that Wnt/β-catenin signaling was rapidly upregulated in response to damage. The upregulation of Wnt/β-catenin signaling corresponded with the peak of cell proliferation in the reparative dentinogenesis process, suggesting that Wnt/β-catenin signaling is mediating a proliferative expansion following damage. Furthermore, proliferating Wnt responsive cells could be detected at the site of damage. Our genetic tracing results indicated that Axin2-expressing cells undergo proliferative expansion following damage and some Axin2-expressing cells differentiate into odontoblast-like cells. At the end of the tracing period, the Axin2-expressing cells were in close association with the dentine bridge and presented long processes that extend into the dentine, a characteristic morphological feature of an odontoblast.

We confirmed that post-mitotic primary odontoblasts express Axin2 and that elevation of the level of Wnt/β-catenin signaling enhances the production of reparative dentine in Axin2^LacZ/LacZ^ mice^22^.

Interestingly, constitutive activation of the Wnt/β-catenin signaling did not promote primary odontoblasts to secrete reactionary dentine, but did enhance reparative dentinogenesis. Blocking Wnt/β-catenin signaling prevented the formation of reparative dentine in response to damage. Specific blocking of the ability of Axin2-expressing cells to release Wnts severely compromised the formation of reparative dentine. This finding indicates that Axin2-expressing cells are acting as their own source of Wnt ligands to drive reparative dentinogenesis via autocrine Wnt/β-catenin signaling. Autocrine Wnt/β-catenin signaling has been shown to be the mechanism by which some regenerating tissues renew. Axin2-expressing cells are responsible for skin and hair follicle renewal and these cells co-express Wnt ligands^17, 20^. Furthermore, we showed that preventing Axin-2 expressing cells from secreting Wnts decreased the expansion of proliferative cells post-damage. This suggests that autocrine Wnt/β-catenin signaling stimulates cell proliferation in response to damage and provides an explanation for why reparative dentinogenesis is impaired in Axin2^CreERT2^; Wls^flox-flox^ mice.

**Figure 6:**
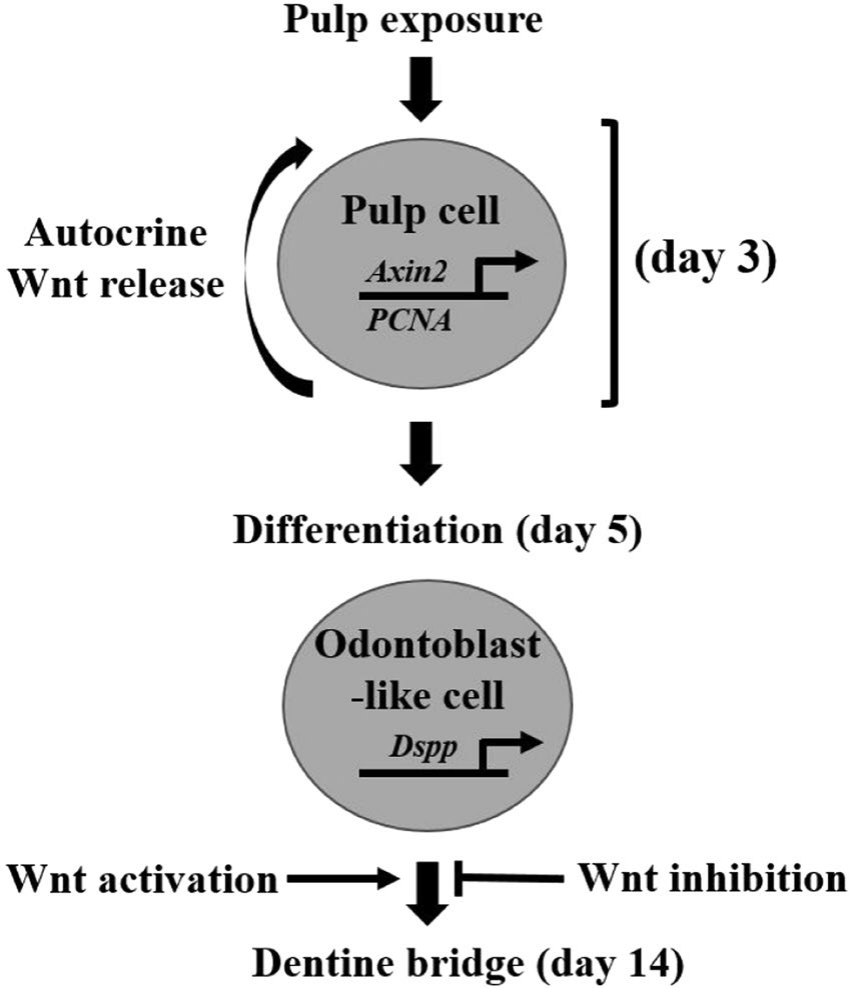
Wnt/β-catenin signaling modulates reparative dentinogenesis. Pulp cells rapidly proliferate in response to tooth damage shown by PCNA staining, with a significant peak in proliferation occurring 3 days post-damage and returning to baseline 14 days post-damage (Fig. 2). New odontoblast-like cells are detected by *DSPP* expression as early as 5 days post-damage and a dentine bridge is seen 14 days post-damage (Fig. 1). Our data shows that pulp exposure first triggers proliferation, followed by odontoblast differentiation and secretion of reparative dentine to form a dentine bridge. Wnt reporter mice (TCF/Lef:H2B-GFP) demonstrated proliferating cells are Wnt responsive 3 days post-damage (Fig. 3). Real-time qPCR analysis of Axin2 expression demonstrated that Axin2 is significantly elevated 3 days post-damage, indicating that the Wnt responsive cells are Axin2 positive ((Fig. 3C), Supplementary Figure 1). Lineage tracing of Axin2 cells in Axin2^CreERT2, Rosa26 mTmG fl/+^ mice demonstrated that these cells undergo a proliferative expansion and differentiate into odontoblast-like cells indicated by their co-expression of Dspp 5 days post-damage, characteristic odontoblast morphology and close association with the dentine bridge 14 days post-damage (Fig. 4). Loss of Wnt signaling in Wls^fl/fl^ mice demonstrated that damaged teeth no longer repair as a dentine bridge is absent 14 days post-damage compared to WT (Fig. 5B). Moreover, specifically deleting Wls in Axin2-expressing cells in Axin2^CreERT2^; Wls^fl/fl^ mice severely impaired dentine bridge formation 14 days post-damage compared to WT (Fig. 5D). This suggests that Axin2 cells are producing their own source of Wnt to modulate their fate in an autocrine manner. Additionally, Wnt signaling is important for damage induced proliferation as the number of proliferating cells are significantly reduced in Axin2^CreERT2^; Wls^fl/fl^ compared to WT at 3 and 5 days post-injury (Fig. 5E).

Our study shows that Wnt/β-catenin signaling is important for the lifespan of primary odontoblasts as well as the generation of new odontoblast-like cells in response to tooth damage. We identify that Axin2 is expressed in odontoblast-like cells and that Axin2-expressing cells may be the source of their own proliferative signals in reparative dentinogenesis (Fig. 6). The role for Wnt/β-catenin signaling in mature primary odontoblasts is unclear. Overexpression of Wnt/β-catenin signaling in primary odontoblasts did not trigger the production of excessive dentine in the absence of damage. This suggests that Wnt/β-catenin signaling is not enhancing the secretory activity of odontoblasts. The ability of Wnt/β-catenin signaling to selectively enhance reparative dentinogenesis is intriguing and may suggest that Wnt/β-catenin signaling is working in synergy with other damage activated signaling pathways (such as signaling molecules sequestered in dentine tubules that are released in response to trauma and injury) to potentiate dentine production^32, 33^. Alternately, Wnt/β-catenin signaling may be increasing the number of odontoblast-like cells, whereas primary odontoblast are unaffected as they are post-mitotic. Further studies to elucidate the dual roles of Wnt/β-catenin signaling in reactionary and reparative dentinogenesis could identify therapeutic strategies that actively promote stem cell driven tooth repair.

## Electronic Supplementary material

Supp data
