# Supplementary material for: Axin2-expressing cells differentiate into reparative odontoblasts via autocrine Wnt/β-catenin signaling in response to tooth damage
Source: Sci Rep. 2017 Jun 8;7:3102. doi: 10.1038/s41598-017-03145-6 (PMC5465208; doi:10.1038/s41598-017-03145-6)
Supplement: Supplementary file 1 — Supp data [file 41598_2017_3145_MOESM1_ESM.doc]

**Axin2-expressing cells differentiate into reparative odontoblasts via autocrine Wnt/β-catenin signaling in response to tooth damage**

Rebecca Babb, Dhivya Chandrasekaran, Vitor Neves, and Paul T Sharpe

**Supplementary Information**

**Figures**

**
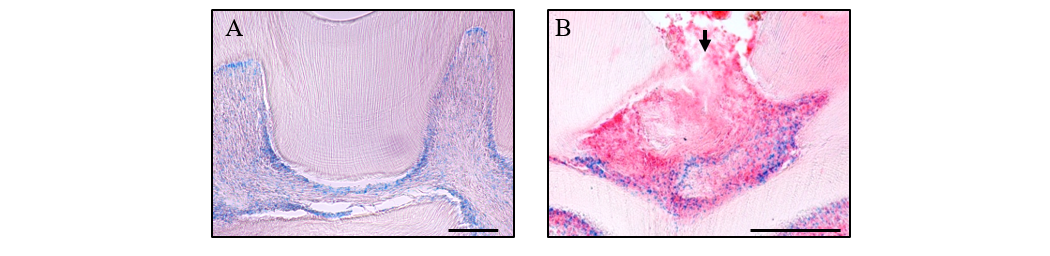
**

**Supplementary Figure S1. Axin2 positive cells are present at the site of tooth damage.** LacZ staining of an undamaged maxillary first molar (A) and a maxillary first molar 3 days after injury from Axin2­­­-LacZ mice (B). Note that odontoblasts located at the periphery of the pulp chamber are positive for Axin2 expression in the undamaged molar. Axin2 positive cells are diffusely scattered under the site of exposure in the damaged molar. Representative sagittal sections are shown from four independent experiments. Scale bars are equivalent to 100 μM and an arrow indicates pulp exposure.


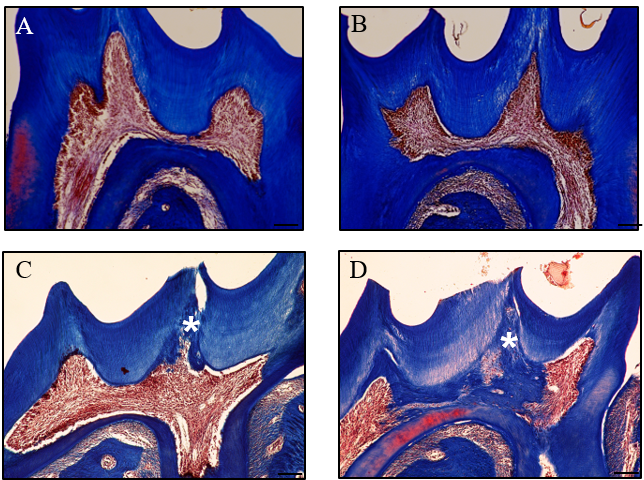


**Supplementary Figure S2. Elevated Axin2 expression enhances repair in damaged molars.** Masson’s Trichrome staining of a WT (A) and Axin2-LacZ/LacZ undamaged superior first molars (B), WT (C) and Axin2-LacZ/LacZ damaged superior first molar (D) 40 days post-damage. Note that undamaged teeth were collected from age matched litter mate mice and not histological differences were observed. Representative sagittal sections are shown from four independent experiment’s. Scale bars are equivalent to 100 μM and an asterisk indicates the formation of a dentine bridge.

**
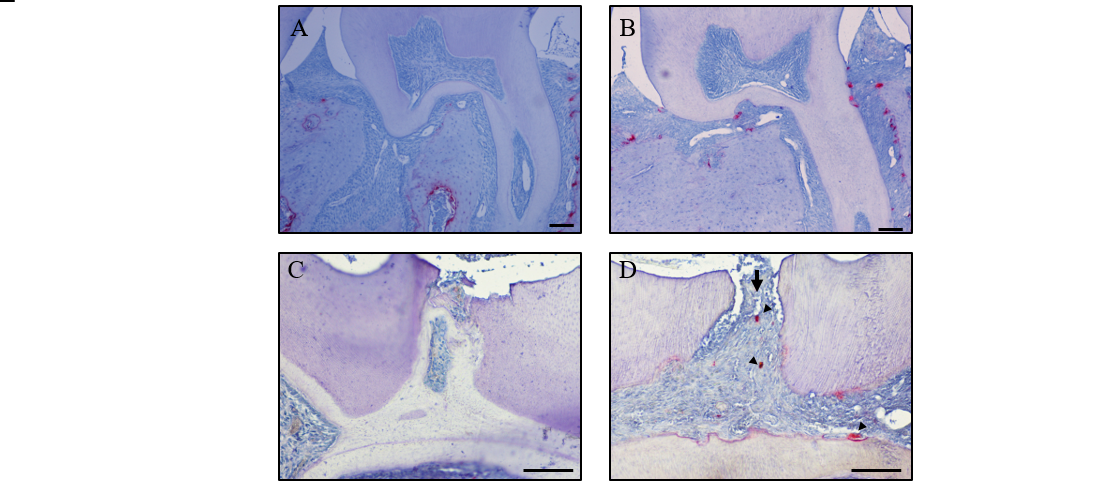
**

**Supplementary Figure S3. Loss of Wnt signaling recruits osteoclasts to the pulp chamber of damaged molars.** TRAP staining of an undamaged molar from a corn oil treated Wlsfl/fl mice(A) and a tamoxifen treated Wlsfl/fl mice (WT) 14 days after administration (B). TRAP staining of a maxillary first molar from a corn oil treated Wlsfl/fl mice(C) and a maxillary first molar from a tamoxifen treated Wlsfl/fl mice (WT) 14 days post-damage (D). Representative sagittal sections are shown from four independent experiments. No osteoclasts were observed in the pulp chamber of damaged WT teeth. Scale bars are equivalent to 100 μM. An arrow indicates exposed pulp, arrow heads indicate TRAP positive osteoclasts and an asterisk indicates the formation of a dentine bridge.

**Supplementary Methods**

**LacZ staining**

Teeth were fixed in 0.4% paraformaldehyde (PFA) for 24-hours at 4˚C, washed with PBS and decalcified in 19% EDTA pH 8 for 4-weeks. Teeth were immersed in 30% sucrose/PBS overnight at 4˚C before embedding in OCT (optimal cutting temperature) using dry ice and ethanol. Sections were cut at a thickness of 12-μm using a cryostat and fixed (0.2% glutaraldehyde, 0.2% NP40, 5 mM EDTA, 2 mM MgCl2 in PBS) then washed in wash buffer (2 mM MgCl2, 0.02% sodium deoxycholate, 0.02% NP40 in PBS). LacZ staining was visualised using a staining solution of 1 mg/ml X-gal substrate (Invitrogen) in wash buffer containing 5 mM potassium ferrocyanide andpotassium ferricyanide at 37ºC for 16-hour. Sections counterstained with nuclear fast red, dehydrated, cleared in Neo-Clear and mounted with Neo-Mount.

**TRAP staining**

Sections of adult teeth were deparaffinised in xylene and rehydrated in graded ethanol. Osteoclasts were identified by staining for tartrate-resistant acid phosphatase (TRAP) activity (Sigma-Aldrich) in 50mM Na-Tartrate, counterstained with hematoxylin and aqueous mounted.
